# A special issue on Bayesian inference: challenges, perspectives and prospects

**DOI:** 10.1098/rsta.2022.0155

**Published:** 2023-05-15

**Authors:** Christian P. Robert, Judith Rousseau

**Affiliations:** ^1^ CEREMADE, University of Paris Dauphine, Paris, France; ^2^ Department of Statistics, University of Warwick, Coventry, UK; ^3^ Department of Statistics, University of Oxford, Oxford, UK

**Keywords:** Bayesian statistics, Bayesian inference, computational statistics, Markov chain Monte Carlo methods, hierarchical Bayes modeling, statistical decision theory

This special issue is dedicated to Sir Adrian Smith, whose contributions to Bayesian analysis have deeply impacted the field (or rather fields) of Bayesian inference, decision theory and statistical computing. When contemplating his past achievements, it is striking to align the emergence of massive advances in these fields with some papers or books of his. For instance, Lindley’s & Smith’s ‘Bayes Estimates for the Linear Model’ (1971) [1], a Read Paper at the Royal Statistical Society, is making the case for the Bayesian analysis of the most standard statistical model, as well as emphasizing the notion of exchangeability that is foundational in Bayesian statistics, and paving the way to the emergence of hierarchical Bayesian modelling. It thus makes a link between the early days of Bruno de Finetti, whose work Adrian Smith translated into English, and the current research in non-parametric and robust statistics. Bernardo’s & Smith’s masterpiece, Bayesian Theory (1994) [2], sets statistical inference within decision- and information-theoretic frameworks in a most elegant and universal manner that could be deemed a Bourbaki volume for Bayesian statistics if this classification endeavour had reached further than pure mathematics. It also emphasizes the central role of hierarchical modelling in the construction of priors, as exemplified in Carlin’s *et al.* ‘Hierarchical Bayesian analysis of changepoint problems’ (1992) [3].

The series of papers published in 1990 by Alan Gelfand & Adrian Smith, esp. ‘Sampling-Based Approaches to Calculating Marginal Densities’ (1990) [4], is overwhelmingly perceived as the birth date of modern Markov chain Monte Carlo (MCMC) methods, as it brought to the whole statistics community (and then quickly wider communities) the realization that MCMC simulation was the sesame to unlock complex modelling issues. The consequences on the adoption of Bayesian modelling by non-specialists are enormous and long-lasting.

Similarly, Gordon’s *et al.* ‘Novel approach to nonlinear/non-Gaussian Bayesian state estimation’ (1992) [5] is considered as the birthplace of sequential Monte Carlo, aka particle filtering, with considerable consequences in tracking, robotics, econometrics and many other fields.

Titterington’s, Smith’s & Makov’s reference book, ‘Statistical Analysis of Finite Mixtures’ (1984) [6] is a precursor in the formalization of heterogeneous data structures, paving the way for the incoming MCMC resolutions like Tanner & Wong (1987) [7], Gelman & King (1990) [8] and Diebolt & Robert (1990) [9]. Denison *et al.*’s book, ‘Bayesian methods for nonlinear classification and regression’ (2002) [10] is another testimony to the influence of Adrian Smith on the field, stressing the emergence of robust and general classification and nonlinear regression methods to analyse complex data, prefiguring in a way the later emergence of machine-learning methods, with the additional Bayesian assessment of uncertainty. It is also bringing forward the capacity of operating Bayesian non-parametric modelling that is now broadly accepted, following a series of papers by Denison *et al.* in the late 1990s [11] like CART and MARS.

Peter Grünwald [12] proposes a decision-based construction of Bayesian inference and uncertainty quantification via e-posteriors that bypasses the precise construction of *a prior* distribution. It is advanced as a more robust form of inference, with guaranteed frequentist properties.

Chris Holmes & Stephen Walker [13] connect exchangeability with Bayesian bootstrap. Exchangeability is at the core of Bayesian thinking, as introduced by de Finetti (1937) [14] and emphasized in Bernardo & Smith (1994) [2]. Their perspective reinforces this position by explaining exchangeability through learning, which is the main argument for pursuing the Bayesian approach to statistics, while connecting with a more classical approach by predicting an infinite population from the current dataset. By extending the notion of Bayesian bootstrap, they reformulate the Bayesian paradigm as purely predictive.

Sonia Petrone [15] also focuses on the predictive aspect of Bayesian inference, making the crucial point that it is a natural way to connect with machine-learning imperatives. She makes the method appear as naturally embedded within non-parametric statistics, as a form of Polva urn sampling, and establishes limit coverage properties that grant frequentist properties to the ensuing Bayesian procedures.

Veronika Rockova & Lizhen Nie [16] brings forward another perspective on the Bayesian bootstrap. The approach there is more likelihood-free in the sense that the sampling model is no longer defined through a likelihood but instead through a loss function as in Bissiri *et al.* (2016) [17]. A strong innovation in this approach is to rely on a machine-learning-based tool, a generative sampler that allows for a generalization of the Bayesian bootstrap of Newton & Raftery (1994) [18] and that brings both computational advantages and theoretical guarantees, including under model mis-specification, a perspective under-studied in the field.

When seeking computational solutions to approximate an essential Bayesian entity, namely the Bayes factor (or the evidence), Arnaud Doucet *et al.* [19] similarly exploit machine-learning tools like variational auto-encoders and normalizing flows to build rich families of approximations in settings where the likelihood function is unavailable. They bring in an annealing perspective to improve further the computational efficiency of these importance sampling methods.

Alicia Carriquiry & Kori Khan [20] build a safer environment for forensic calibration studies by resorting to hierarchical Bayes modelling, investigated by Adrian Smith in his early career, and borrowing from small area estimation, thus bringing a quantitative evaluation of the imprecision in using this tool for reporting error rates.

Matthew Stephens [21] contributes a broader perspective to the volume by connecting Bayesian principles with non-Bayesian procedures, like confidence intervals and *p*-values, thus connecting with Peter Grünwald e-values. He also advocates considering alternative approaches for faster or more robust resolutions.

Sylvia Früwirth-Schnatter [22] broadens the range of shrinkage priors in connection with spike-and-slab priors used in variable selection and factor analysis. She reveals new connections with Bayesian non-parametrics and exchangeability. Her computational aspects are also most relevant for the theme of this volume since they involve MCMC and data augmentation, where Adrian Smith's contributions have been fundamental.

Sara Wade [23] advocates for a Bayesian approach to cluster analysis, of which Adrian Smith has been an early researcher when working on mixtures of distributions (Titterington *et al.* 1984 [6]). She brings another connection with the Gibbs priors of Bissiri *et al.* (2016) [17].

Kerrie Mengersen & her co-authors [24] also offer a very broad perspective on the opportunities and challenges of applied Bayesian analysis, including design (with links to decision theory), data sources and citizen science, federated learning and data privacy, with MCMC consequences, implicit models and ABC, and model transfer. All these points are illustrated by diverse and exciting case studies.

Richard Nickl & co-authors [25] study hindrances to MCMC convergence in high dimensions, reconnecting with the early days when MCMC and simulated annealing were created in parallel and phase transition sounded like an epiphenomenon. The notion of entropy wells is quite appealing in demonstrating that convergence issues can arise with MCMC even in the case of unimodal (if not log-concave) targets. This paper provides further arguments against local Monte Carlo methods and possibly in favour of new schemes like PDMPs although none is included here. For a specific Gaussian tensor model, the posterior concentrates exponentially fast (with the dimension) over a small region of the space, inducing an equally exponential time for ‘small step’ MCMC algorithms to converge. This includes both unadjusted and Metropolis-adjusted Langevin algorithms.

Peter Müller *et al.* [26] open a new perspective on the Bayesian exploitation of complex data sources like medical records. They demonstrate how Bayesian non-parametrics can be used in that context, with consequence on drug trials. The approach relies on MCMC methods. The paper also relates to Adrian Smith's work on mixtures and Bayesian non-parametrics.

Sumio Watanabe [27] presents his original perspective on Bayesian information criteria (BIC), within the context of cross-validation, including new results on the asymptotic behaviour of Bayes factors, new types of losses and BICs. The approach defended therein also allows for an assessment of prior distributions and a novel form of empirical Bayes analysis by optimizing hyperparameters.

Beatrice Franzolini & co-authors [28] study an integer-supported zero-inflated model called the hurdle model, with the addition of a clustering mechanism, connecting with Sara Wade’s article [24]. The work builds on earlier advances on finite mixtures with random number of components, to bring higher computational efficiency in the Bayesian analysis of the model than when using non-parametric alternatives.

## Data Availability

This article has no additional data.
